# The Influence of Different Hematite (α-Fe_2_O_3_) Particles on the Thermal, Optical, Mechanical, and Barrier Properties of LDPE/Hematite Composites

**DOI:** 10.3390/ma16020706

**Published:** 2023-01-11

**Authors:** Ana Peršić, Nina Popov, Ljerka Kratofil Krehula, Stjepko Krehula

**Affiliations:** 1 Faculty of Chemical Engineering and Technology, University of Zagreb, Marulićev Trg 19, 10000 Zagreb, Croatia; 2 Division of Materials Chemistry, Ruđer Bošković Institute, Bijenička 54, 10000 Zagreb, Croatia

**Keywords:** polymer composites, low-density polyethylene, hematite, particle shape, particle size, thermal stability, UV blocking, mechanical properties, barrier properties

## Abstract

There is an increasing need to develop new polymer composites with improved properties compared to conventional pure polymer materials. This work aims to develop composites of low-density polyethylene (LDPE) and iron oxide hematite particles. For this purpose, different types of hematite particles with well-defined shapes and narrow size distributions were synthesized: HC2 sample with pseudocubic hematite particles of an average diameter of 1020 nm, HE1 sample with ellipsoidal hematite particles of an average diameter of 533 nm, and HS1 sample with spherical hematite particles of an average diameter of 168 nm. The mass fractions of hematite in the composites were 0.25%, 0.5%, and 1%. Prepared LDPE/hematite composites were characterized by thermogravimetric analysis (TGA), Fourier transform infrared spectroscopy (FTIR), and diffuse reflectance ultraviolet-visible-near infrared (UV-Vis-NIR) spectroscopy. The mechanical and barrier properties were also studied. The obtained results showed that all prepared composites have improved properties compared to the pure LDPE, especially the composites with pseudocubic hematite particles of well-defined shapes. The results of this study indicate that LDPE/hematite composites can be promising materials for a wide range of applications, especially as packaging materials where improved thermal and mechanical properties as well as resistance to ultraviolet (UV) irradiation are required.

## 1. Introduction

Nowadays, there is an increasing need to develop new polymer composites with improved or additional properties compared to conventional pure polymer materials. Among the many different types of polymer composites, a significant group represents those with metal oxide particles embedded in different polymer matrices. In many cases, such a combination can have a beneficial effect on different properties and offer different application possibilities of the resulting polymer composites due to improved material characteristics: thermal stability, mechanical strength, light absorption, barrier properties, or antibacterial characteristics.

For example, the preparation of polymer composites with iron oxide hematite (α-Fe_2_O_3_) is highly desirable due to the low cost, non-toxicity, thermal stability, corrosion resistance, and strong UV absorption of hematite, which can lead to significantly improved material properties. From previous research on polymer composites with hematite, it is known that such materials have various improved properties: greater thermal stability [1], better mechanical properties [2,3], better corrosion resistance [4], greater absorption of electromagnetic radiation [5], and better sensing properties [6]. Due to the high thermal stability and UV-blocking effect of hematite [7], polymer composites with hematite have potential for use as packaging materials [3] and materials with UV-blocking and flame-retardant properties [8]. The aforementioned studies published in the scientific literature dealt with the preparation and characterization of composites with hematite using different polymer matrices: polystyrene [1,2], polyethylene [9], polyurethane [4], polyacrylonitrile [10], polyaniline [6], poly(vinyl-pyrrolidone) [11], and polycaprolactone [12]. However, systematic research on the influence of the size and shape of hematite particles on the properties of composites with a polymer matrix has not yet been published. It is important to point out that the sizes and shapes of metal oxide particles can also have significant influences on the properties of the composite in addition to the type of metal oxide and its mass fraction in the composite.

This work tends to develop low-density polyethylene/hematite composites prepared with hematite particles of different shapes and sizes. Such types of composites have not yet been thoroughly studied but have the potential to reveal improved properties compared to pure low-density polyethylene polymer. Polyethylene is one of the most commonly used polymer materials and has a wide range of applications, primarily as packaging for food or cosmetic products. It is a material of low price and very good processability and health safety characteristics. Likewise, hematite is a non-toxic, chemically and thermally stable, and low-cost metal oxide. In this work, the influences of hematite particles of different sizes and shapes on the various properties of prepared polymer composites were studied. The properties of thus prepared hematite particles and LDPE/hematite composites were determined by different instrumental techniques. It is important to highlight that the preparation of the composites with non-aggregated hematite particles and their fine dispersion in polyethylene matrix is a demanding task. In our preliminary study, we successfully prepared LDPE/hematite composites using two hematite samples of different particle sizes and investigated their properties [13].

The aim of this work was to synthesize uniform hematite particles of different sizes and shapes using the hydrothermal synthesis method by adjusting the experimental conditions (reactant concentrations, additives, reaction times, etc.). Particle sizes and shapes are important factors that affect various properties of hematite samples [14,15,16,17,18]. Uniformity of particles is very important and desirable because samples with uniform particles have better defined properties that are easier to interpret and compare than samples with poor uniformity. The influence of such prepared hematite particles on the properties of polymer composites was thoroughly studied to propose a preparation method and composition of low-density polyethylene/hematite composites with the best possible properties for their potential application as new and improved materials. The influence of the shapes and sizes of hematite particles on the properties of polymer composites, to our best knowledge, has not yet been published. This work combines the synthesis of hematite particles of different shapes and sizes as well as the preparation of polymer composites, which has the potential to reveal new and significant results.

## 2. Materials and Methods

### 2.1. Preparation of the Hematite Particles and LDPE/Hematite Composites

Low-density polyethylene/hematite composites were prepared from LDPE polymer granulate (Dow Chemical) and hydrothermally synthesized hematite particles (samples HC2, HE1, and HS1). Hematite particles were prepared from concentrated FeCl_3_ and NaOH aqueous solutions by procedures based on the method proposed by Sugimoto et al. [19,20]. To prepare pseudocubic hematite particles (sample HC2), 2.5 mL of H_2_O and 47.5 mL of 6 M NaOH aqueous solution were slowly added to 50 mL of 2 M FeCl_3_ aqueous solution in a polypropylene (PP) bottle under strong magnetic stirring. The obtained suspension was heated at 100 °C in a laboratory oven for four days. To prepare ellipsoidal hematite particles (sample HE1), 3.5 mL of H_2_O, 1.5 mL of 1 M Na_2_SO_4_ aqueous solution, and 45 mL of 6 M NaOH aqueous solution were slowly added to 50 mL of 1.95 M FeCl_3_ aqueous solution in a PP bottle under strong magnetic stirring. The obtained suspension was heated at 100 °C for six days. To prepare spherical hematite particles (sample HS1), 50 mL of 6 M NaOH aqueous solution was slowly added to 50 mL of 2 M FeCl_3_ aqueous solution in a PP bottle under strong magnetic stirring. The obtained suspension was heated at 100 °C for eight days. Precipitated hematite particles in all three samples were washed (using double distilled water and centrifuge) and dried at 60 °C in air.

**Figure 1:**
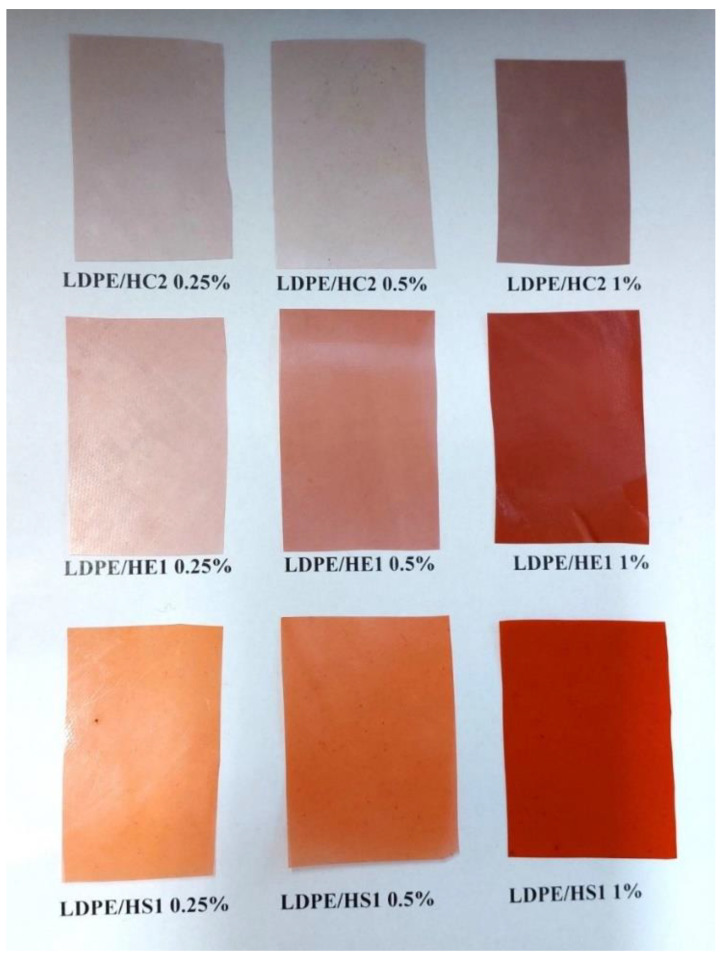
Prepared foils of LDPE/hematite composites.

The LDPE/hematite composites, named LDPE/HC2, LDPE/HE1, and LDPE/HS1, were prepared by mixing in a Brabender kneader at a temperature of 180 °C over a period of 3 min with a speed of kneading of 45 rpm. The contents of hematite in the samples were 0.25, 0.5, and 1% (Table 1). For further characterization, the obtained composite materials were shaped into the foils (Figure 1) and plates by pressing using a hydraulic press (Dake model 44-226) at a temperature of 190 °C.

### 2.2. Characterization

A JEOL JSM-7000F field-emission scanning electron microscope (FE-SEM) was used for the observation of hematite particles morphology. The acceleration voltage of 10 kV was applied. The particle size distribution was determined by selecting 100 clearly visible hematite particles on the corresponding SEM images and measuring their sizes (diameters). A Malvern Panalytical Empyrean diffractometer (CuKα radiation) was used to record diffraction patterns of the prepared hematite samples. The thermal stability of LDPE and the obtained polymer composites was determined by the thermogravimetric analyzer, TA Instruments Q500. Mass specimens of 10 mg were analyzed in the nitrogen stream at a heating rate of 10 °C/min in the temperature range from 25 to 800 °C. Pure LDPE and the prepared composites were characterized by attenuated total reflectance (ATR) Fourier transform infrared spectroscopy (FTIR) on a Perkin Elmer Spectrum One FTIR spectrometer in the range from 4000 to 650 cm^−1^ with a resolution of 4 cm^−1^. The characterization was repeated four times for each sample to ensure replicate data was produced. Mechanical properties were determined on a Zwick 1445 universal device. Samples were 100 mm long and 10 mm wide (~1 mm thick). The stretching speed was 50 mm/min. Diffuse reflectance UV-Vis-NIR spectra of polymer composite films were recorded at 20 °C using a Shimadzu UV-3600 UV-Vis-NIR spectrophotometer with an integrating sphere. Barium sulfate (Nacalai Tesque) was used as a reference material. Determination of water vapor permeability of all samples was carried out using Herfeld’s apparatus, which consists of a glass container and a metal lid with a circular hole (diameter of 36 mm). For measurement of water vapor permeability, 50 mL of water was poured into the glass container. The studied sample foil (diameter of 55 mm) was placed under the metal lid, and the lid was closed. The glass container was put in a desiccator with 97% sulphuric acid. The weight of the glass container with the specimen and the water was determined at the beginning of the measurement and after 24 and 48 h.

## 3. Results and Discussion

### 3.1. Properties of Hematite Particles

**Figure 2:**
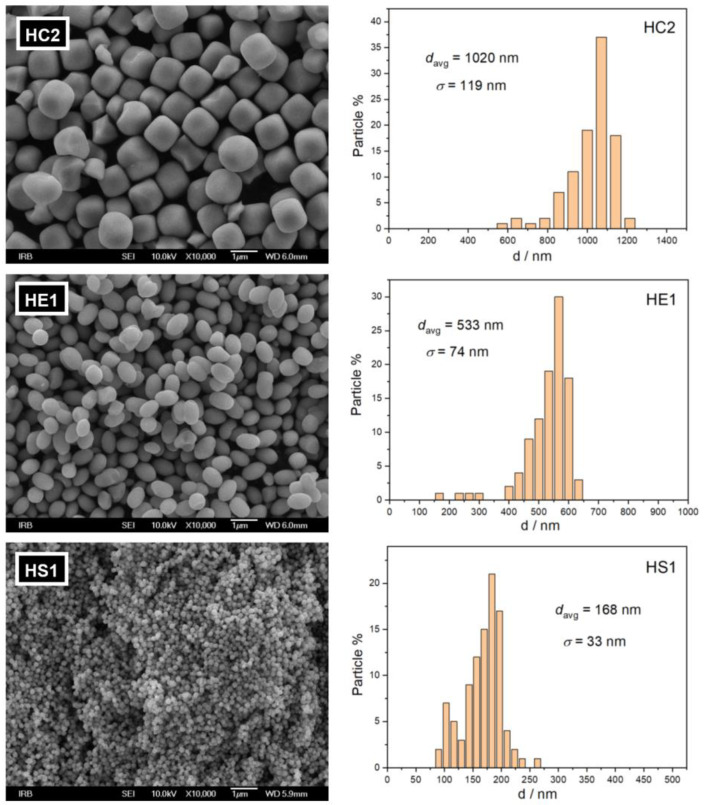
FE-SEM images of synthesized hematite samples, HC2, HE1, and HS1, with corresponding particle size (diameter) distributions.

The sizes and shapes of the synthesized hematite particles were observed using scanning electron microscopy. Pseudocubic, ellipsoidal, and spherical hematite particles of average diameters of 1020 ± 119 nm, 533 ± 74 nm, and 168 ± 33 nm are present in samples HC2, HE1, and HS1, respectively (Figure 2).

**Figure 3:**
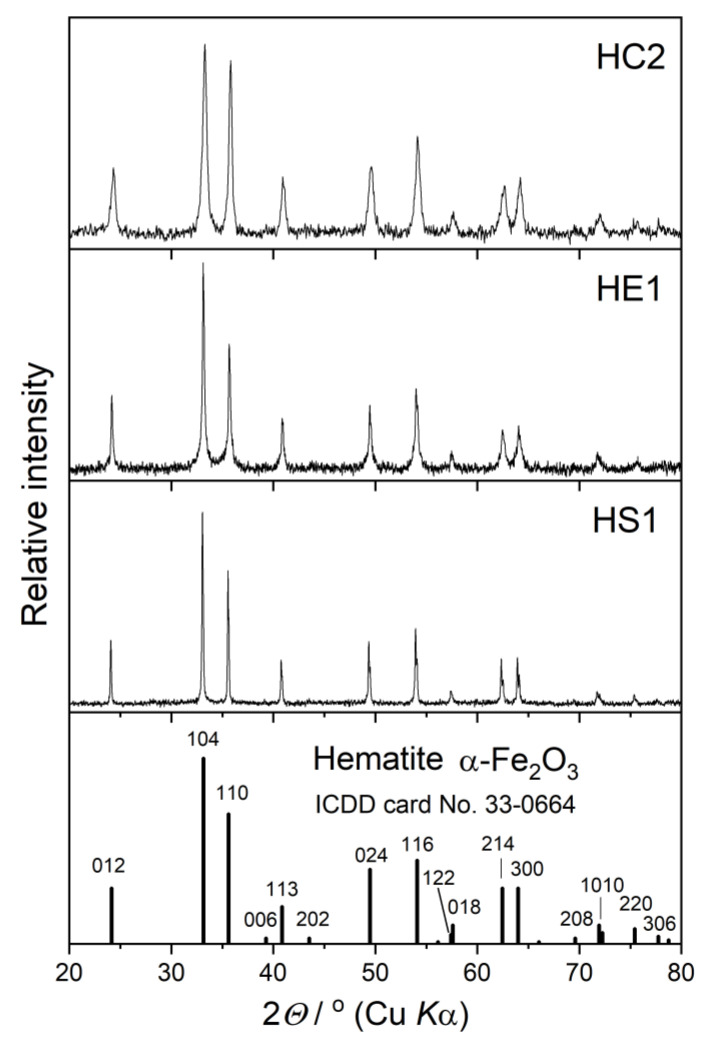
PXRD patterns of synthesized hematite samples, HC2, HE1, and HS1, with the positions and intensities of the diffraction lines given in the ICDD card of hematite.

The powder X-ray diffraction (PXRD) patterns of the synthesized samples are shown in Figure 3. All patterns match well with the standard hematite diffraction pattern (ICDD PDF card No. 33-0664) with no additional diffraction lines of other crystalline phases. A significant difference in the width of the diffraction lines in these three PXRD patterns suggests differences in the crystallite sizes of the prepared hematite samples. The average crystallite size in the prepared samples, estimated using the Scherrer equation [21], is inversely proportional to the particle size: 22 nm for sample HC2, 54 nm for sample HE1, and 90 nm for sample HS1. According to these results, the largest, pseudocubic particles (sample HC2) consist of thousands of nanosized subcrystals, and the ellipsoidal particles (sample HE1) consist of hundreds of subcrystals, while the smallest, spherical particles are composed of only a few subunits. The sizes of the hematite particles and crystallites had significant influence on various properties (optical, electronic, magnetic, photocatalytic, etc.) [14,15,16,17,18].

### 3.2. Thermogravimetric Analysis

The properties of the polymer composites highly depend on the type of polymer matrix used, the concentration, size and shape of filler particles, as well as on the interactions between the polymer matrix and the filler particles. Different types of metal oxides, used as fillers in polymer composites, may improve the overall properties and stability of some types of polymers. The aim of this work was to study the influences of the shapes and sizes of hematite particles on the properties of polymer composites prepared with a polyethylene matrix. Due to the lack of information and the absence of a systematic study of the use of hematite in LDPE matrix, especially hematite particles of different shapes and sizes, this work could contribute to such knowledge. It is important to point out that the thermal properties of polymer composites are extremely important during their use, and it is known that some types of polymer composites can show significant thermal stability with the use of a hematite filler. For instance, polystyrene/hematite composites show good improvement in thermal stability compared to pure polystyrene [1,22]. In the mentioned study where the content of hematite in the polystyrene matrix was 3.6 wt%, the shift of the polystyrene/hematite decomposition to a temperature about 100 °C higher compared to the decomposition of the pure polymer polystyrene was observed [1].

**Figure 4:**
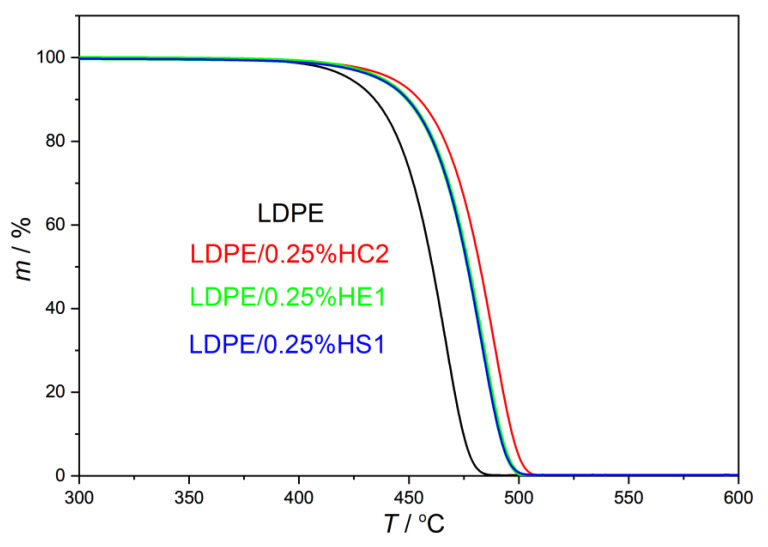
TG curves of prepared LDPE/hematite composites containing 0.25% hematite particles.

**Figure 5:**
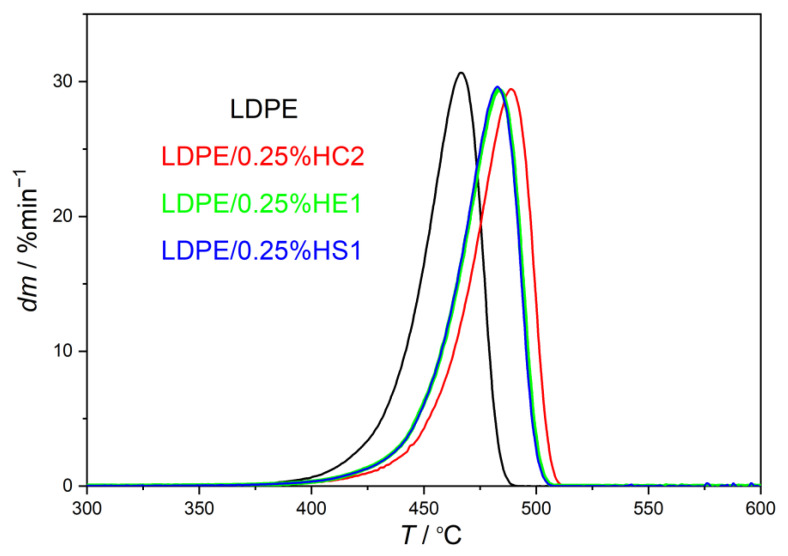
DTG curves of prepared LDPE/hematite composites containing 0.25% hematite particles.

In the present study, the thermal stability of LDPE/hematite composites was studied by thermogravimetric analysis (TGA), and the results are presented in Table 2 and in Figure 4 and Figure 5. The aim of this characterization was to compare the thermal stability of LDPE/hematite composites with the thermal stability of the pure LDPE polymer.

The initial decomposition temperature of the samples (*T*_95%_) is studied to compare the beginnings of thermal decomposition of the samples. It represents the temperature where 5% of the sample is decomposed and 95% of the sample remained. The results, presented in Table 2 and Figure 4 and Figure 5, show that all studied composite samples have higher *T*_95%_ than the pure LDPE. The increase in such stability is the consequence of the hematite presence in composites, which contributes to the thermal resistance of the LDPE. The highest value of *T*_95%_ is obtained for the sample LDPE/0.25%HC2 (442.92 °C), which shifted even more than 20 °C toward a higher temperature compared to the pure LDPE polymer (422.60 °C). It can be explained by the presumed changed molecular mobility of polymer chains, which are adsorbed on the surface of hematite particles in the composite. Restricted motions of polymer chains attached to the surface of hematite particles cause better stability of composites compared to the pure LDPE polymer [22]. Furthermore, there is no significant difference between the start of the thermal decomposition when the higher content of the filler is used. The content of the filler of only 0.25% causes the quite strong shift at the beginning of thermal decomposition to the higher temperatures for all types of hematite (HC2, HE1, and HS1). It can be supported by the fact that the small size of the filler particles due to their high surface-to-bulk ratio can considerably improve thermal properties of the polymer matrix even when a very low filler content is used [1].

Furthermore, the obtained results show that the LDPE polymer is thermally degraded in one step due to the presence of one degradation maximum, which is the temperature of maximum rate of decomposition (*T*_max_). All LDPE/hematite composites also degrade in one step but at higher temperatures. That proves the improvement of the thermal stability, using hematite as a filler, for all studied samples compared to the pure LDPE. LPDE composites with hematite HC2 (pseudocubic hematite particles, average size of about 1020 ± 119 nm) show the most significant shift of *T*_max_ to higher temperatures. For LDPE/0.25%HC2 composite, *T*_max_ is higher than *T*_max_ of pure LDPE by as much as 21.30 °C.

It can be concluded that all studied types and contents of hematite in the LDPE matrix improve the thermal stability of LDPE. Even a very low content of hematite (0.25%) significantly improves the thermal resistance of LDPE.

### 3.3. FT-IR Spectroscopy

**Figure 6:**
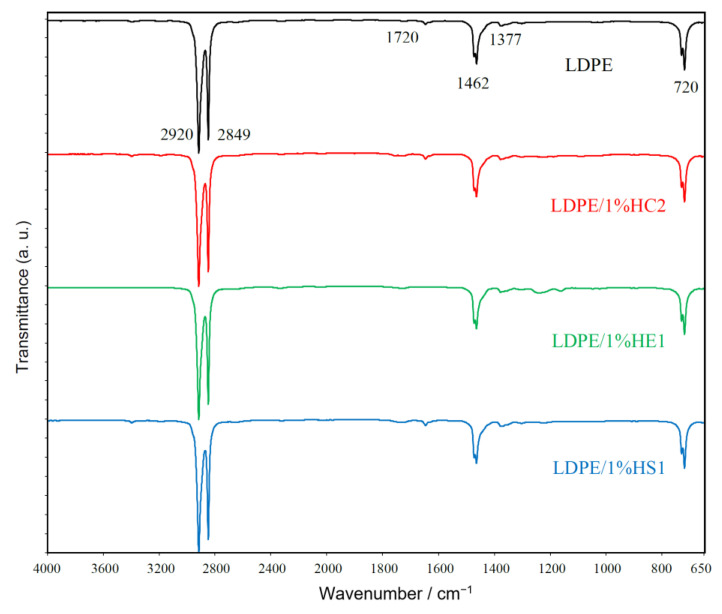
FT-IR spectra of LDPE and prepared LDPE/hematite composites.

Figure 6 presents FTIR spectra of LDPE polymer and LDPE/1%HC2, LDPE/1%HE1, and LDPE/1%HS1 composite samples. The spectra reveal characteristic vibrations for polyethylene polymer: peaks of strong intensity for CH_2_ asymmetric stretching at 2920 cm^−1^ and CH_2_ symmetric stretching at 2849 cm^−1^; a peak at 1462 cm^−1^ which corresponds to bending deformation and a peak at 720 cm^−1^, which corresponds to rocking deformation. A weak peak at 1377 cm^−1^, which represents CH_3_ symmetric deformation [23,24,25], is also observed.

There are no significant differences between the FTIR spectra of pure LDPE and LDPE/hematite composites. Characteristic peaks for hematite are not observed due to the spectral range of the measurement from 4000 to 650 cm^−1^, while hematite IR bands could be revealed only below 650 cm^−1^ [26].

The peak of very weak intensity for carbonyl groups at about 1720 cm^−1^ was observed for all studied samples. It is caused by the low degree of polyethylene degradation [27], which may always occur during processing due the raised temperature, pressures, and the presence of oxygen. It is very important to mention that there is no increase of the peak for carbonyl groups in composite samples compared to pure LDPE. For this reason, it can be concluded that hematite does not cause polyethylene degradation during preparation of the composites. Furthermore, there is no occurrence of other characteristic groups (for example vinyl groups at 909 cm^−1^), which may be formed during polyethylene degradation [27].

### 3.4. UV-Vis-NIR Spectroscopy

**Figure 7:**
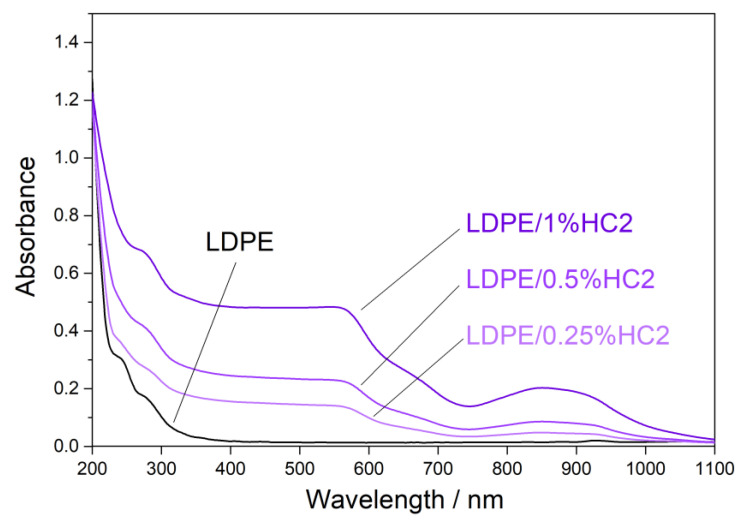
Absorbance UV-Vis-NIR spectra (obtained from diffuse reflectance) of LDPE and LDPE/HC2 composites.

**Figure 8:**
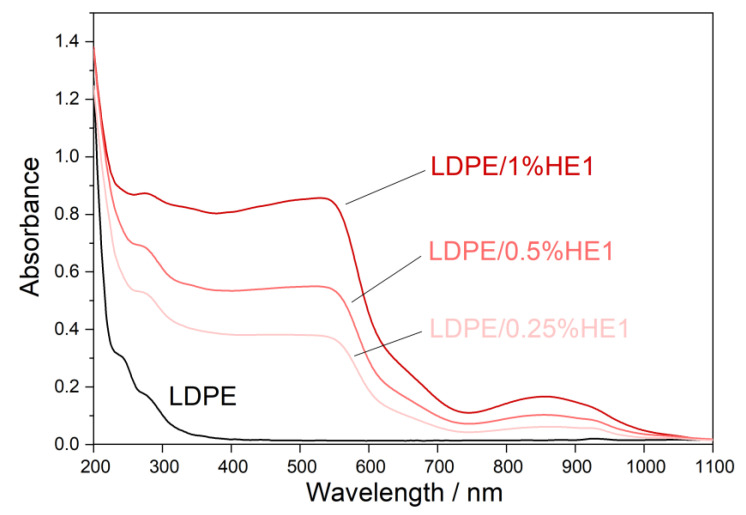
Absorbance UV-Vis-NIR spectra (obtained from diffuse reflectance) of LDPE and LDPE/HE1 composites.

**Figure 9:**
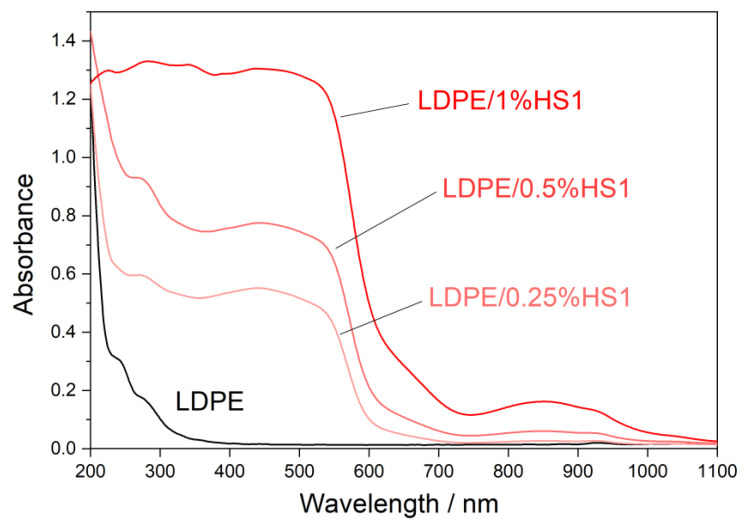
Absorbance UV-Vis-NIR spectra (obtained from diffuse reflectance) of LDPE and LDPE/HS1 composites.

Ultraviolet (UV) light highly influences the properties of polymer materials during their transportation, storage, shelf-life, and use. Colorless and colored polymer materials absorb UV radiation, which is of sufficient energy to break bonds in polymers in a process of photodegradation. The UV degradation in polymers is initiated by the presence of some substances, which absorb UV light, such as some types of additives and catalyst residues [28]. To keep polymer products resistant to UV degradation for a longer period of time, new UV protective polymer materials can be formulated. It is possible to prepare some types of polymer composites with certain types of fillers, which will provide the polymer material with a UV-blocking property. Figure 7, Figure 8 and Figure 9 present UV-Vis-NIR spectra of polymer composites compared to pure LDPE. The absorption intensity for all LDPE/hematite composites is higher than for LDPE. All studied composites show absorption in UV and in the visible region. The highest absorption intensity was observed for LDPE/HS1 composites due to the smallest particle size of hematite HS1. This observation can be explained by the large surface of the hematite particles, which increases their absorption intensity and UV-blocking property. In general, due to the presence of hematite, the prepared LDPE/hematite composite materials have UV protective properties, i.e., strong UV light absorption. Absorbed light hematite re-emits mainly as heat [29]. Finally, it can be concluded that the incorporation of hematite into the LDPE matrix ensures efficient protection against the harmful effects of UV light.

### 3.5. Mechanical Properties

**Figure 10:**
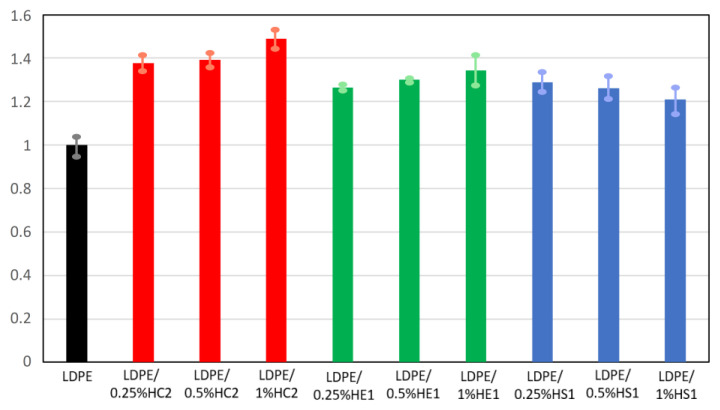
Relative tensile strength of LDPE and LDPE/hematite composites.

**Figure 11:**
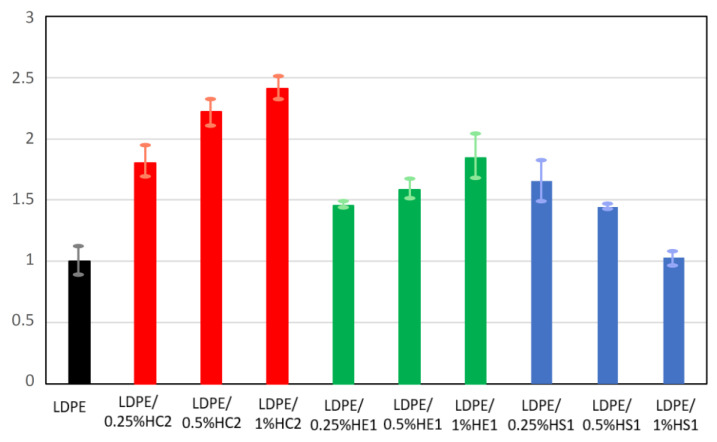
Relative elongation at break of LDPE and LDPE/hematite composites.

The mechanical properties of some types of polymers can be significantly improved using hematite as a filler where it contributes to the enhancement of these properties. It is also known that mechanical properties depend on filler content and its aggregation as well as on polymer viscosity [3,30]. The results of the mechanical properties of pure LDPE and LDPE/hematite composites are presented in Figure 10 and Figure 11, expressed as relative tensile strength and relative elongation at break. The tensile strength and elongation at break of the prepared composites are expressed in relation to the tensile strength and elongation at break of the pure LDPE. Pure LDPE is used as a reference material and its tensile strength and elongation at break are expressed as value 1. The unit for measured tensile strength was Nmm^−2^, while for elongation at break it was %.

An improvement in mechanical properties was observed for all tested composite samples compared to the pure LDPE. For some composite samples, this improvement was significant. The tensile strength of all composites was at least 20% higher than the tensile strength of the neat LDPE polymer. The LDPE/HC2 samples had the highest tensile strength, which for the LDPE/1%HC2 composite was 48.9% higher than the tensile strength of LDPE. Furthermore, the elongation at break for the composite samples was significantly improved. The highest values were again obtained for LDPE/HC2 samples, where the increase was as much as 80.6, 122.4, and 140.9% for LDPE/0.25%HC2, LDPE/0.5%HC2, and LDPE/1%HC2, respectively. Such results indicate a good miscibility of LDPE polymer and all types of hematite filler. It is known that polymer composites with enhanced mechanical properties can be prepared using a suitable mixture design and mixing procedure [30]. Our preparation procedure includes mixing the polymer and filler before melting. In this way, the polymer and the filler are mixed and brought together even before mixing in the melt. Finally, during the preparation of the composites by melting, the filler becomes uniformly distributed in the LDPE matrix, the polymer chains are attached to the surface of hematite particles, and stability of the composites is good. The results showed that the mechanical properties of the composites were significantly improved even with a low content of filler (0.25%), presumably due to the good dispersion of the low amount of filler in the polymer. For the smallest used hematite particles (HS1), which had an average size of 168 ± 33 nm, deterioration of mechanical properties was observed with an increasing amount of filler. This can be explained by the possible filler agglomeration when its higher quantity is present in the polymer matrix. It is important to point out that this work is focused on the preparation of different hematite types with controlled particle shapes and sizes and to the study of their influences on the LDPE matrix. Therefore, it can be concluded that the most significant influence on the composites’ mechanical properties has hematite type HC2 due to its specific pseudocubic geometry, which allows excellent interactions and adhesion of filler and matrix.

### 3.6. Barrier Properties

If there is a need for storing of wet products in polymer packaging, it is very important to avoid water loss from the packaging. For this reason, polymer material can be modified with the aim to decrease water vapor permeability. The permeability of composites is affected by the type of polymer matrix, the mobility of polymer chains, and the degree of polymer crystallinity. The type of filler and the degree of its dispersion in the polymer matrix also have an influence on the permeability of the composite [31]. The presence of dispersed nanoparticles with a large surface area in the polymer matrix can increase the barrier properties of polymer nanocomposites. Homogeneous dispersion of the filler creates a tortuous path in the polymer matrix and increases the length of the diffusion path for water loss [32]. The results of water vapor permeability for LDPE and LDPE/hematite composites are given in Table 3.

The results showed that most of the quantity of water from the studied samples is lost during the first 24 h. During the next 24 h, the loss of water is very low. Namely, the filler absorbs water on its surface, and at the same time, acts as a barrier to water evaporation. It is assumed that during the first 24 h the equilibrium is reached, the hematite particles become saturated with water, their volume increases, the water evaporation path is prolonged, and the water further evaporates very slowly through the polymer film. It was also observed that all studied samples have considerably lower water permeability than pure LDPE. For the LDPE/HC2 and LDPE/HE1 composites, water permeability decreased with increasing filler content. The opposite situation was observed in LDPE/HS1 composites, probably due to the already mentioned agglomeration of small HS1 particles when the filler is not evenly distributed in the polymer matrix. In general, the permeability properties of polymer materials can be used to predict product shelf-life, which can be extended by using a specific type of protective packaging [33]. It can be concluded that the prepared LDPE/hematite composites have the potential to be effectively used as packaging materials for the prevention of moisture loss.

## 4. Conclusions

Based on the results presented and discussed above, it can be concluded that the LDPE/hematite composites developed in this work have considerably improved properties compared to the pure LDPE polymer. The addition of hydrothermally synthesized hematite particles of different shapes and sizes significantly improved the overall properties of LDPE. Each synthesized hematite type (HC2, HE1, and HS1) is of uniform shape and size. The thermal stability of the LDPE/hematite composites is higher compared to the pure LDPE polymer. A significant shift in LDPE decomposition toward higher temperatures was found due to the incorporation of hematite filler into the LDPE matrix. The mechanical properties are also significantly improved. All prepared composites also showed improved UV-blocking properties and significantly reduced water permeability compared to the pure LDPE. Even a very small amount of hematite filler (0.25%) significantly improved the overall properties of LDPE. Pseudocubic hematite particles showed the greatest influence on the properties of the composites. Finally, it is very important to point out that this work combines the synthesis of hematite particles of different shapes and sizes with the preparation of polymer composites, revealing significant results. According to the highly improved overall properties compared to the pure LDPE polymer, all studied LDPE/hematite composites can be promising materials for various applications, especially as packaging materials.

## Figures and Tables

**Table 1:** Composition of prepared samples.

Sample	LDPE (wt%)	Hematite (wt%)
LDPE	100	0.00
LDPE/0.25%HC2	99.75	0.25
LDPE/0.5%HC2	99.50	0.50
LDPE/1%HC2	99.00	1.00
LDPE/0.25%HE1	99.75	0.25
LDPE/0.5%HE1	99.50	0.50
LDPE/1%HE1	99.00	1.00
LDPE/0.25%HS1	99.75	0.25
LDPE/0.5%HS1	99.50	0.50
LDPE/1%HS1	99.00	1.00

**Table 2:** Initial decomposition temperature of the samples (*T*_95%_) and the temperature of the maximum rate of decomposition (*T*_max_).

Sample	*T*_95%_ (°C)	*T*_max_ (°C)
LDPE	422.60	467.07
LDPE/0.25%HC2	442.92	488.37
LDPE/0.5%HC2	442.68	487.87
LDPE/1%HC2	439.32	484.29
LDPE/0.25%HE1	437.87	482.60
LDPE/0.5%HE1	435.71	481.87
LDPE/1%HE1	434.99	483.32
LDPE/0.25%HS1	436.94	482.14
LDPE/0.5%HS1	438.38	483.57
LDPE/1%HS1	436.23	479.27

**Table 3:** Water vapor permeability of LDPE and LDPE/hematite composites.

Sample	Water Loss after 24 h/g	Total Water Loss (after 48 h)/g
LDPE	0.275	0.299
LDPE/0.25%HC2	0.014	0.018
LDPE/0.5%HC2	0.012	0.014
LDPE/1%HC2	0.009	0.011
LDPE/0.25%HE1	0.015	0.019
LDPE/0.5%HE1	0.012	0.017
LDPE/1%HE1	0.010	0.014
LDPE/0.25%HS1	0.015	0.021
LDPE/0.5%HS1	0.020	0.031
LDPE/1%HS1	0.024	0.046

## Data Availability

The data that support the findings of this study are available from the corresponding authors (L.K.K and S.K.) upon reasonable request.

## References

[1] Marinović-Cincović M., Šaponjić Z.V., Djoković V., Milonjić S.K., Nedeljković J.M. (2006). The influence of hematite nano-crystals on the thermal stability of polystyrene. Polym. Degrad. Stab..

[2] Djoković V., Nedeljković J.M. (2000). Stress relaxation in hematite nanoparticles-polystyrene composites. Macromol. Rapid Commun..

[3] Kausar A. (2020). Polymeric materials filled with hematite nanoparticle: Current state and prospective application. Polym. Plast. Technol. Mater..

[4] Palimi M.J., Rostami M., Mahdavian M., Ramezanzadeh B. (2015). A study on the corrosion inhibition properties of silane-modified Fe_2_O_3_ nanoparticle on mild steel and its effect on the anticorrosion properties of the polyurethane coating. J. Coat. Technol. Res..

[5] Chen D., Quan H., Huang Z., Luo S., Luo X., Deng F., Jiang H., Zeng G. (2014). Electromagnetic and microwave absorbing properties of RGO@hematite core–shell nanostructure/PVDF composites. Compos. Sci. Technol..

[6] Bora M.A., Adhav P.B., Diwate B.B., Pawar D.S., Dallavalle S., Chabukswar V.V. (2019). Room temperature operating sensitive and reproducible ammonia sensor based on PANI/hematite nanocomposite. Polym. Plast. Technol. Mater..

[7] Truffault L., Choquenet B., Konstantinov K., Devers T., Couteau C., Coiffard L.J.M. (2011). Synthesis of nano-hematite for possible use in sunscreens. J. Nanosci. Nanotechnol..

[8] Camlibel N.O., Arik B., Avinc O., Yavas A. (2018). Antibacterial, UV protection, flame retardancy and coloration properties of cotton fabrics coated with polyacrylate polymer containing various iron ores. J. Text. Inst..

[9] Yurkov G.Y., Gubin S.P., Pankratov D.A., Koksharov Y.A., Kozinkin A.V., Spichkin Y.I., Nedoseikina T.I., Pirog I.V., Vlasenko V.G. (2002). Iron(III) oxide nanoparticles in a polyethylene matrix. Inorg. Mater..

[10] Greenstein K.E., Myung N.V., Parkin G.F., Cwiertny D.M. (2019). Performance comparison of hematite (α-Fe_2_O_3_)-polymer composite and core-shell nanofibers as point-of-use filtration platforms for metal sequestration. Water Res..

[11] Matysiak W., Tański T., Zaborowska M. (2019). Electrospinning process and characterization of PVP/hematite nanofibers. IOP Conf. Ser. Mater. Sci. Eng..

[12] Mensah E.E., Abbas Z., Azis R.S., Ibrahim N.A., Khamis A.M., Abdalhadi D.M. (2020). Complex permittivity and power loss characteristics of α-Fe_2_O_3_/polycaprolactone (PCL) nanocomposites: Effect of recycled α-Fe_2_O_3_ nanofiller. Heliyon.

[13] Peršić A., Popov N., Štefanović E., Krehula S., Govorčin Bajsić E., Leskovac M., Kratofil Krehula L. The influence of the size of hematite particles on the properties of polyethylene/hematite composites. Proceedings of the NANOCON Conference Proceedings—International Conference on Nanomaterials.

[14] Chernyshova I.V., Ponnurangam S., Somasundaran P. (2010). On the origin of an unusual dependence of (bio)chemical reactivity of ferric hydroxides on nanoparticle size. Phys. Chem. Chem. Phys..

[15] Townsend T.K., Sabio E.M., Browning N.D., Osterloh F.E. (2011). Photocatalytic water oxidation with suspended alpha-Fe_2_O_3_ particles—Effects of nanoscaling. Energy Environ. Sci..

[16] Demarchis L., Sordello F., Minella M., Minero C. (2015). Tailored properties of hematite particles with different size and shape. Dyes Pigm..

[17] Luo X., Song S., Ma M., Wang Y., Zhou Y., Zhang Y. (2019). Effect of particle size on flotation performance of hematite. Physicochem. Probl. Miner. Process..

[18] Meijer J.M., Rossi L. (2021). Preparation, properties, and applications of magnetic hematite microparticles. Soft Matter.

[19] Sugimoto T., Sakata K. (1992). Preparation of monodisperse pseudocubic α-Fe_2_O_3_ particles from condensed ferric hydroxide gel. J. Colloid Interface Sci..

[20] Sugimoto T., Wang Y., Itoh H., Muramatsu A. (1998). Systematic control of size, shape and internal structure of monodisperse α-Fe_2_O_3_ particles. Colloids Surf. A Physicochem. Eng. Asp..

[21] Klug H.P., Alexander L.E. (1974). X-ray Diffraction Procedures: For Polycrystalline and Amorphous Materials.

[22] Kuljanin J., Marinović-Cincović M., Zec S., Čomor M.I., Nedeljković J.M. (2003). Influence of Fe_2_O_3_-filler on the thermal properties of polystyrene. J. Mater. Sci. Lett..

[23] Gulmine J.V., Janissek J.V., Heise H.M., Akcelrud L. (2002). Polyethylene characterization by FTIR. Polym. Test..

[24] Smith B.C. (2011). Fundamentals of Fourier Transform Infrared Spectroscopy.

[25] Chao Y., Jianming Z., Deyan S., Shouke Y. (2006). Structure characterization of melt drawn polyethylene ultrathin films. Chin. Sci. Bull..

[26] Wang Y., Muramatsu A., Sugimoto T. (1998). FTIR analysis of well-defined α-Fe_2_O_3_ particles. Colloids Surf. A Physicochem. Eng. Asp..

[27] Scott G. (1990). Mechanisms of Polymer Degradation and Stabilisation.

[28] Andrady A.L. (2003). Plastics and the Environment.

[29] Cheremisinoff N.P. (1997). Handbook of Engineering Polymeric Materials.

[30] Gencel O., Brostow W., Martinez-Barrera G., Gok M.S. (2012). Mechanical Properties of Polymer Concretes Containing Different Amount of Hematite or Colemanite. Polimery.

[31] Johansson F., Leufvén A., Ackermann P., Tagerstand M., Ohesson T. (1995). Food Packaging Polymer as Barrier against Aroma Vapour and Oxygen in Fat or Humid Environments. Food and Packaging Materials: Chemical Interactions.

[32] Kausar A. (2020). A review of high performance polymer nanocomposites for packaging applications in electronics and food industries. J. Plast. Film Sheeting.

[33] Hrnjak Murgić Z., Rešček A., Ptiček Siročić A., Kratofil Krehula L., Katančić Z. (2015). Nanoparticles in Active Polymer Food Packaging.

